# MSRB3 antioxidant activity is necessary for inner ear cuticular plate structure and hair bundle integrity

**DOI:** 10.1242/dmm.052194

**Published:** 2025-08-19

**Authors:** Gowri Nayak, Elodie M. Richard, Byung Cheon Lee, Gavin P. Riordan, Inna A. Belyantseva, Bruno Manta, Thomas B. Friedman, Vadim N. Gladyshev, Saima Riazuddin

**Affiliations:** ^1^ Department of Developmental Biology, Cincinnati Children's Hospital Medical Center, Cincinnati, OH 45229, USA; ^2^ Department of Otorhinolaryngology Head and Neck Surgery, School of Medicine, University of Maryland, Baltimore, MD 21201, USA; ^3^ Division of Genetics, Department of Medicine, Brigham and Women's Hospital, Harvard Medical School, Boston, MA 02115, USA; ^4^ College of Life Sciences and Biotechnology, Korea University, Seoul 02841, Republic of Korea; ^5^ Broad Institute, Cambridge, MA 02142, USA; ^6^ Laboratory of Molecular Genetics, National Institute on Deafness and Other Communication Disorders, NIH, Bethesda, MD 20892, USA; ^7^ Department of Molecular Biology and Biochemistry, School of Medicine, University of Maryland, Baltimore, MD 21201, USA

**Keywords:** MSRB3, Methionine sulfoxide reductases, Deafness, DFNB74

## Abstract

Methionine sulfoxide reductases (MSRs) are enzymes responsible for catalyzing the reduction of methionine sulfoxides. We previously demonstrated that variants in human *MSRB3*, an MSR family member, are associated with profound autosomal recessive prelingual non-syndromic deafness, DFNB74. To better understand the role of MSRB3 in the auditory pathway, we generated complete *Msrb3* gene knockout mice. The *Msrb3*-deficient mice showed profound deafness by postnatal day 16, which was accompanied by morphological abnormalities including altered stereocilia bundle shape and cuticular plate degeneration, followed by hair cell apoptotic death. Although the absence of MSRB3 primarily affected the actin cytoskeleton, rootlets were present, and the localization of major F-actin stereocilia-core proteins was unaltered. Biochemical assays demonstrated that wild-type MSRB3, but not MSRB3 harboring p.Cys89Gly, the same variant reported for DFNB74, can repolymerize oxidized actin. Consistent with these results, we observed a decreased ratio of reduced/total actin in the inner ears of *Msrb3* knockout mice. These data suggest a protective role for MSRB3 in the maintenance and maturation of stereocilia and hair cells, a conserved mechanism aimed at maintaining actin redox dynamics in these sensory cells.

## Teaser

**Summary:** Our study suggests a protective role for MSRB3 in the maintenance and maturation of mouse stereocilia and hair cells, a conserved mechanism aimed at maintaining actin redox dynamics in these sensory cells.

## INTRODUCTION

The cochlea is the receptor organ for hearing and consists of rows of sensory and supporting cells that together comprise the organ of Corti. The sensory hair cells perceive sound-induced vibrations through actin-rich structures called the stereociliary bundles on the apical surface of sensory hair cells. Mechanical displacements of the stereociliary bundles are transduced into electrical signals that are then transmitted to the brain. Hair cells, like all other cell types, are susceptible to oxidative stress-induced damage and apoptosis that occur as a result of noise-trauma, ototoxicity or aging (Huang et al., 2000). Reactive oxygen species that are released during hair cell activity and metabolism are dealt with by built-in antioxidant mechanisms, failure of which can lead to hearing loss (Ahmed et al., 2011; Shafique et al., 2014; Richard et al., 2019; Alqudah et al., 2018; Kwon et al., 2014).

Surface-exposed methionine residues in proteins are susceptible to reactive oxygen species that can oxidize them to methionine sulfoxide residues, resulting in loss or altered biological activity of the protein (Levine et al., 1996; Keck, 1996; Brot and Weissbach, 1983; Vogt, 1995). Methionine sulfoxide reductases are enzymes that catalyze the reduction of methionine sulfoxide to methionine found as the free form and also incorporated into proteins (Brot and Weissbach, 1983; Hoshi and Heinemann, 2001; Weissbach et al., 2002; Kim and Gladyshev, 2004a). These enzymes are stereospecific, wherein MSRA is directed to the S diastereomer, methionine-S*-*sulfoxide, while the MSRB family targets methionine-R*-*sulfoxides (Sharov et al., 1999; Moskovitz et al., 2000, 2002; Etienne et al., 2003). The cellular distribution of these enzymes varies. In humans, MSRA and MSRB1 localize to the mitochondria and cytosol, MSRB2 localizes to the mitochondria, and MSRB3 isoforms A and B localize to the endoplasmic reticulum and the mitochondria, respectively (Vougier et al., 2003; Kim and Gladyshev, 2004b). Variants in *MSRB3* were reported to underlie autosomal recessive prelingual non-syndromic deafness, DFNB74, and the protein was shown to localize predominantly to the sensory hair cells in the cochlea (Ahmed et al., 2011; Shafique et al., 2014; Richard et al., 2019; Kwon et al., 2014). *Msrb3* last exon deletion mutant mice and zebrafish morphants have been developed, which exhibit profound hearing loss without vestibular dysfunction, recapitulating the hearing deficit observed in humans (Kwon et al., 2014; Shen et al., 2015). In both models, the sensory epithelia show progressive stereociliary degeneration and, ultimately, hair cell apoptosis. Exogeneous expression of MSRB3, using *in utero* injection of adeno-associated virus particles into embryonic day (E)12.5 mouse embryos, as well as injection of human *MSRB3* isoform A mRNA into zebrafish larvae at the one-cell stage, was able to rescue the hearing deficit as well as the stereocilia and hair cells defects (Shen et al., 2015; Kim et al., 2016). Although these models show the pathogenicity of MSRB3 functional deficit and its impact on the auditory pathway, no molecular mechanisms have been proposed to explain the observed phenotype.

The current study investigated the inner ear phenotype of a mutant mouse in which all the coding exons of *Msrb3* were deleted and the functional consequences of the absence of the encoded redox protein in the cochlea. Loss of MSRB3 resulted in morphological abnormalities in the inner ear hair cells, including altered stereocilia bundle shape and cuticular plate degeneration, followed by hair cell apoptotic death, which stem from increased oxidative damage to actin molecules in cochlear hair cells. This study highlights the importance of an endogenous redox mechanism in the inner ear for maturation and maintenance of the hair bundle, which is necessary for the mechanotransduction of sound.

## RESULTS

### MSRB3 localizes to hair cells of the organ of Corti

**Fig. 1.**
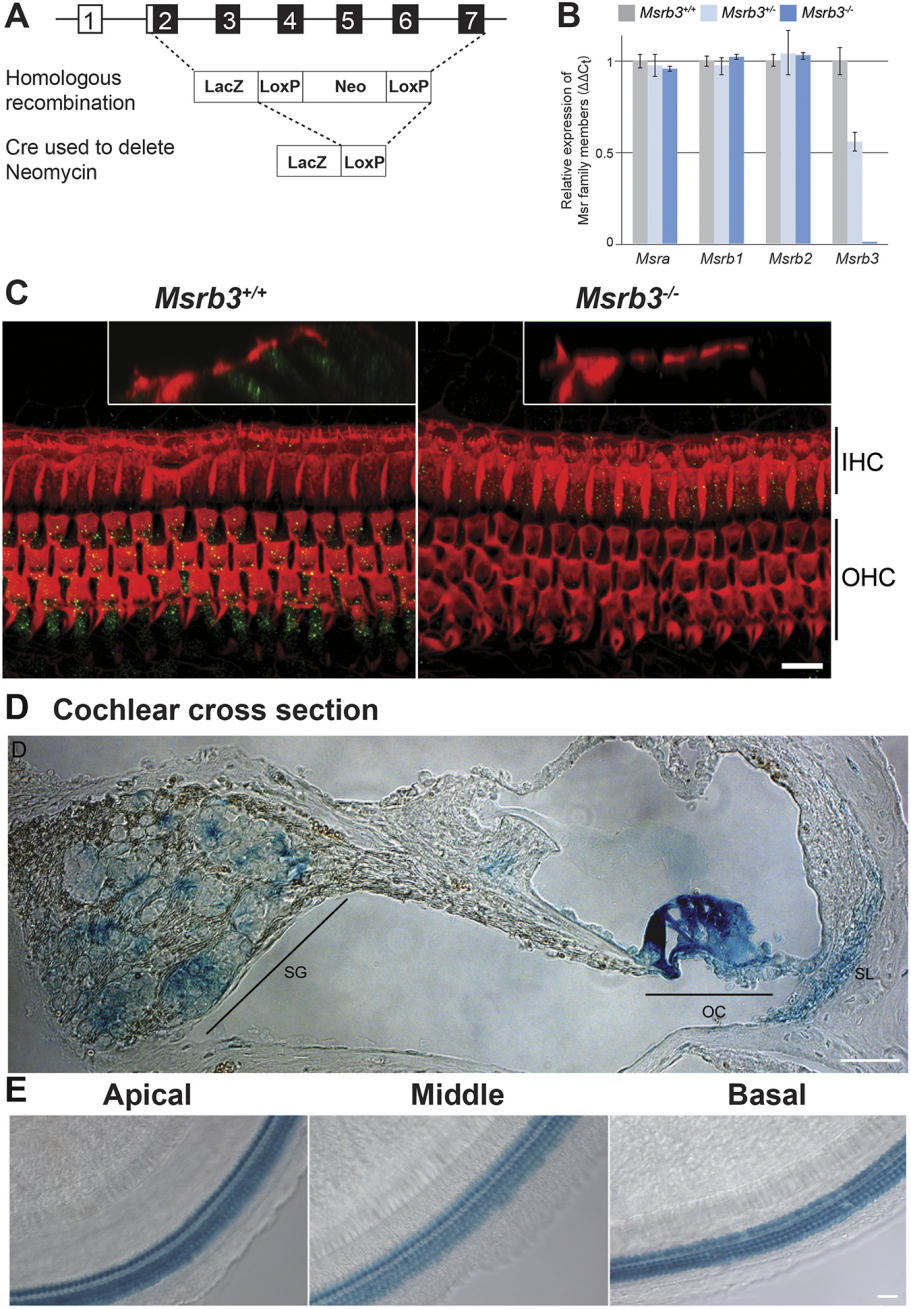
**Generation and validation of *Msrb3* knockout mice.** (A) Schematic representation of generation of *Msrb3^−/−^* mice. Exons 2 to 7 of *Msrb3* were replaced by homologous recombination with a LacZ and a Neomycin (Neo) cassette. The Neo resistance cassette, flanked by LoxP sites, was removed by crossing the mice with mice expressing a Cre recombinase gene [C57BL/6-Tg(Zp3-cre93Knw/J); zona pellucida 3 (*Zp3*) gene]. (B) Quantification of MSR family member expression, normalized against *Gapdh*, in the cochleae of *Msrb3^−/−^* mutant mice and their control littermates (*Msrb3^+/+^* and *Msrb3^+/−^*) at postnatal day (P)0. As anticipated, *Msrb3* expression is reduced in heterozygous mice and undetectable in homozygous mutant mice. No variation of expression was observed in any other genes of the MSR family (means±s.e.m.). (C) Maximum-intensity projections of confocal *z*-stacks of whole-mount cochleae at P16, labeled with anti-MSRB3 antibody (green) and phalloidin (red), are shown. Insets in both images show a *y-z* projection of the organ of Corti's epithelium. Whereas in wild-type *Msrb3^+/+^* mice (left), MSRB3 protein (green) is localized in the cytoplasm of the outer (OHC) and inner (IHC) hair cells, no immunolabeling was detected in *Msrb3^−/−^* mutant mice (right). Scale bar: 10 µm. (D,E) X-galactosidase staining was performed on *Msrb3^+/−^* mice to document *Msrb3* promoter activity in the organ of Corti from P6 mice. *Msrb3* is expressed in hair cells throughout the cochlear turns along with the spiral ganglion (SG) and spiral ligament (SL) regions. OC, organ of Corti. Scale bars: 15 µm.

The *Msrb3* full gene knockout mouse was recovered from a cryopreserved strain of KOMP/VelociGene (Fig. 1A). To excise the Neomycin (Neo) cassette, we crossed the mice acquired from VelociGene with a mouse line expressing the Cre recombinase under the *Zp3* promoter [C57BL/6-Tg(Zp3-cre)93Knw/J] (Yancopoulos, 2003). After Neo cassette removal, loss of MSRB3 expression was confirmed by quantitative PCR and immunolabeling (Fig. 1B,C). Capitalizing on the insertion of a LacZ reporter cassette downstream of the *Msrb3* promoter, we assessed the spatial and temporal expression pattern of *Msrb3* in the inner ear. *Msrb3* expression was noted specifically in hair cells of the cochlear sensory epithelium from early postnatal time points until 2 months of age, the oldest time point studied here (Fig. 1D,E). Reporter expression was also observed in the spiral ganglion and the spiral ligament (Fig. 1D).

### The *Msrb3* knockout mouse has early-onset profound deafness

**Fig. 2.**
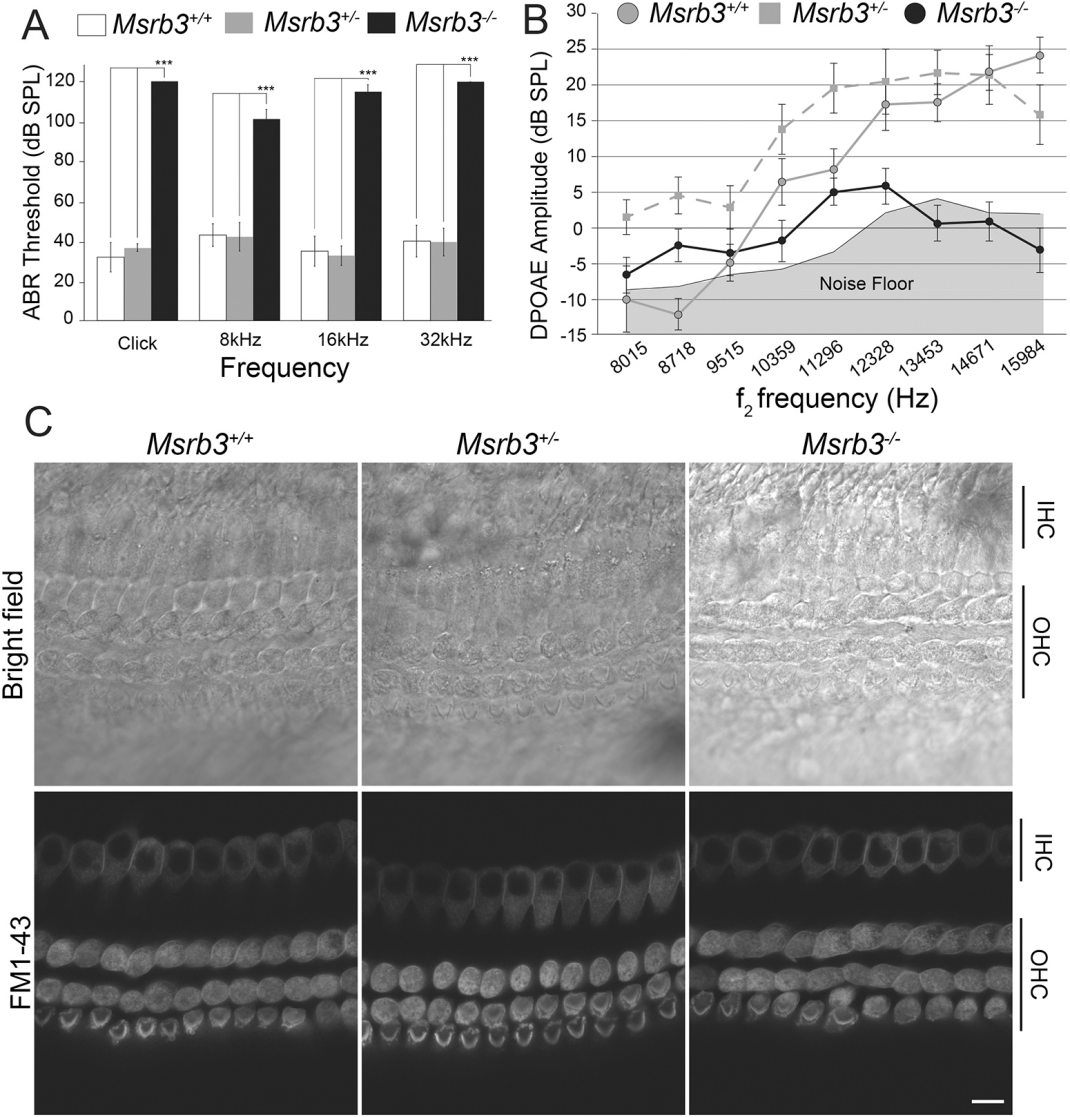
***Msrb3* mutant mice are profoundly deaf by P16 with likely intact mechanoelectrical transducer channel function.** (A) Averaged auditory brainstem response (ABR) thresholds of *Msrb3^−/−^* mice (*n*=9) and their control littermates [*Msrb3^+/−^* (*n*=9) and *Msrb3^+/+^* (*n*=7)] at P16 in response to broadband click stimuli and 8, 16 and 32 kHz tone bursts, with sound pressure level (SPL) of 0 to 120 dB. The *Msrb3^−/−^* mice are profoundly deaf and showed statistically significantly (***P<0.001, two-way ANOVA followed by Bonferroni's test) elevated thresholds for all the tested frequencies compared to their control littermates (mean±s.e.m.). (B) Distortion product otoacoustic emission (DPOAE) amplitudes of *Msrb3^−/−^* (black circles, *n*=9), *Msrb3^+/−^* (gray squares, *n*=9) and *Msrb3^+/+^* (gray circles, *n*=7) at P16, represented as a function of *f2* stimulus frequencies. *Msrb3^−/−^* mice showed reduced to no responses, with values close to the noise floor, suggesting that their OHCs at this age are non-functional (mean±s.e.m.). (C) Mechanotransduction was assessed by FM1-43 dye uptake in *Msrb3^−/−^* and control littermate mouse explants at P5. No apparent difference in the uptake of FM1-43 dye was observed between the mutant and the control mice. IHC, inner hair cell; OHC, outer hair cell. Scale bar: 5 µm.

The cochlear function of *Msrb3^−/−^* and control (*Msrb3^+/+^*, *Msrb3^+/−^*) mice was evaluated by measuring auditory brainstem responses (ABRs) using broadband click and tone-burst sounds at postnatal day (P)16, the earliest age at which ABRs can be successfully recorded. Whereas *Msrb3^+/+^* and *Msrb3^+/−^* mice had normal and indistinguishable ABR thresholds, *Msrb3^−/−^* mice did not respond to click or tone-burst stimuli of even a 100 dB sound pressure level (SPL), indicating that they are profoundly deaf (Fig. 2A). Distortion product otoacoustic emissions (DPOAEs), which represent the outer hair cell (OHC) function, were found to be reduced to no responses in the mutant mice, although the responses were often not discernible from the noise floor (Fig. 2B). The ABR and DPOAE assessments suggested that the absence of MSRB3 leads to cochlear deficiencies that result in hearing loss. Next, mechanotransduction of cochlear hair cells was evaluated at P5 for control and *Msrb3^−/−^* mutant mice. We observed no differences in the uptake of the channel-permeable fluorescent styryl dye FM1-43 (Fig. 2C), indicating that *Msrb3* deficiency is unlikely to directly affect mechanotransduction at P5.

### Loss of MSRB3 results in early postnatal developmental defects in the organ of Corti

**Fig. 3.**
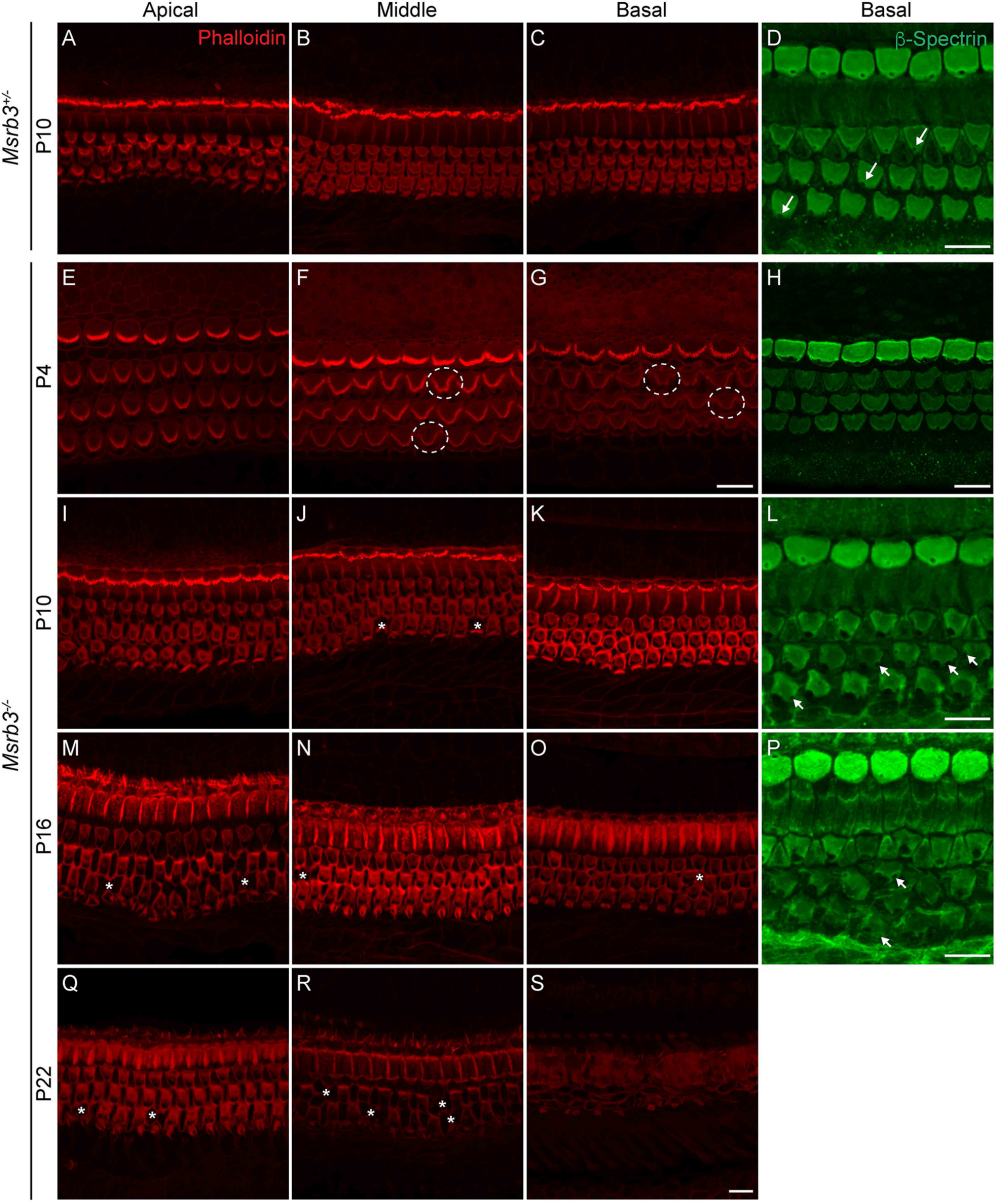
**Organ of Corti hair cells of *Msrb3* mutant mice showed abnormal stereociliary bundles, cuticular plate defects and progressive hair cells degeneration.** Maximum-intensity projections of confocal *z*-stacks of whole-mount cochleae labeled with anti-β-spectrin (green) and phalloidin (red) are shown. (A-C) Representative images from the apical (A), middle (B) and basal (C) turns of the organ of Corti of a control mouse (*Msrb3^+/−^*) at P10. (D) Representative image of hair cells cuticular plates labeled with β-spectrin from the basal turn of the organ of Corti of a control mouse (*Msrb3^+/−^*) at P10. Arrows indicate ‘spectrin-free’ zones in the kinocilial region. (E-S) Representative images of the organ of Corti from the three turns of the cochleae of *Msrb3^−/−^* mice at P4 (E-H), P10 (I-L), P16 (M-P) and P22 (Q-S). H, L, and P show representative images of hair cells cuticular plates labeled with β-spectrin from the basal turn of the organ of Corti of a mutant mouse (*Msrb3^−/−^*) at P4, P10 and P16, respectively. Compared to control mice, *Msrb3^−/−^* mice showed abnormal stereocilia bundles, starting at P4, in middle (F) and basal (G) turns (dashed line circles). By P10, some signs of hair cell loss are visible (J, asterisks) associated with abnormal holes (arrows) in the spectrin-labeled cuticular plate (L). Similar spectrin-free holes were observed at P16 (P, arrows). The hair cell loss worsened rapidly, and, by P22, all hair cells of the basal turn were degenerated (S), and both middle (R) and apical (O) turns showed some hair cell loss (asterisks). Scale bars: 10 µm. Scale bar in S applies to A-C,I-R. Scale bar in G applies to E and F. Asterisks indicate missing hair cells.

**Fig. 4.**
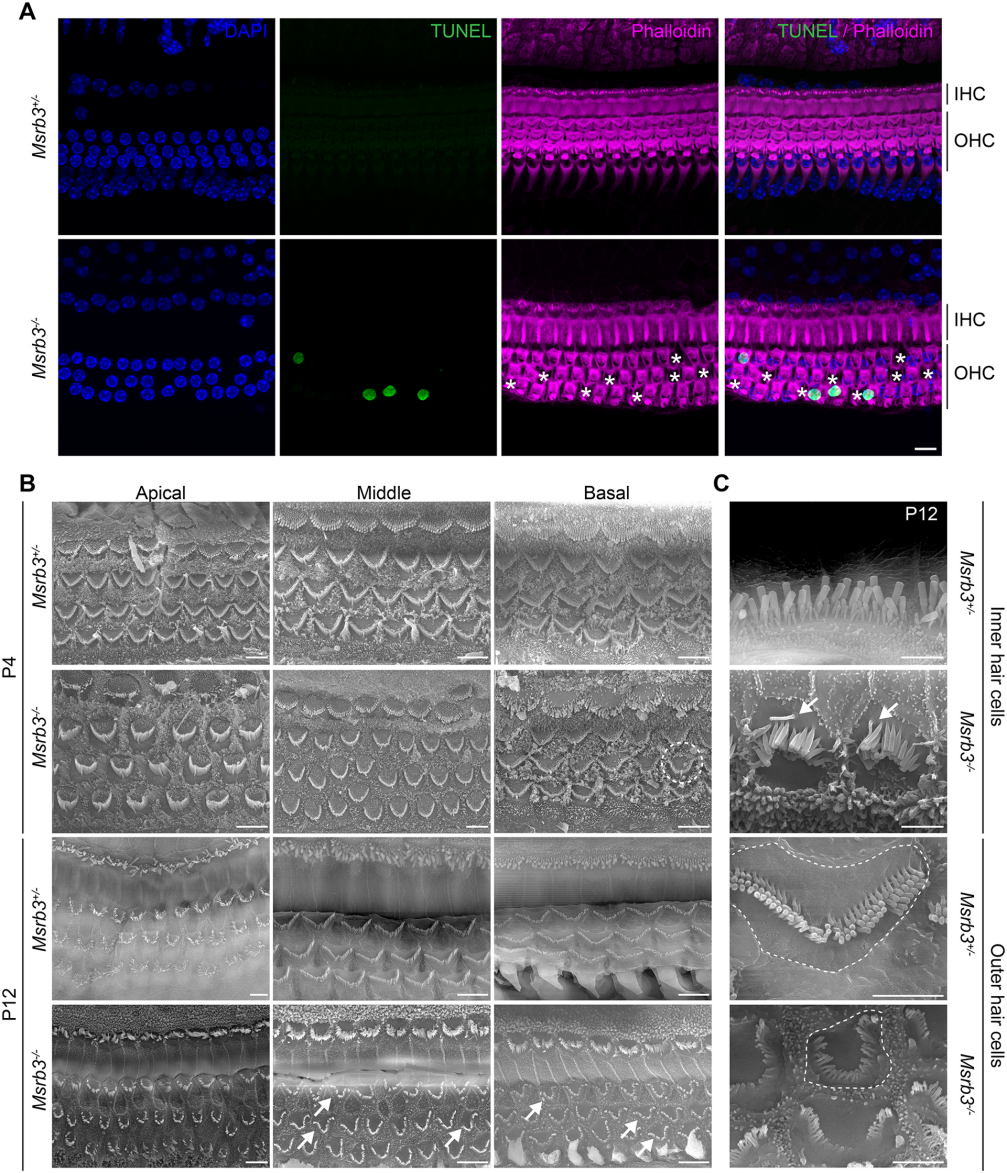
**Loss of MSRB3 induces apoptosis and disrupts the stereocilia bundle morphology.** (A) TUNEL assay (green) was performed on whole-mount cochleae of *Msrb3^+/−^* control and *Msrb3^−/−^* mice at P20. Nuclei were highlighted by DAPI (blue), and actin filaments were stained using phalloidin (magenta). Only hair cells in *Msrb3^−/−^* mutant mice were TUNEL positive. Asterisks indicate missing hair cells. Scale bar: 5 µm (applies to all panels). (B) Scanning electron micrographs of the organ of Corti from the three turns of the cochleae of *Msrb3^−/−^* and the heterozygous control mice at P4 and P12. Starting in the basal turn, the stereocilia bundles of the *Msrb3^−/−^* mice lost the characteristic ‘V’ shape seen in the controls as early as P4, showing instead an ‘omega’ shape (dashed line circle). By P12, most of the stereocilia bundles in the middle and basal turns of the cochlea exhibit an omega shape (arrows). Scale bars: 5 µm. (C) High-magnification images of representative IHC and OHC stereocilia bundle morphology of *Msrb3^−/−^* and control mice at P12. The dashed lines around the edges of OHCs in C highlight the more rounded appearance of the *Msrb3^−/−^* mouse stereociliary bundle compared to the *Msrb3^+/−^* stereociliary bundle. Arrows indicate omega-shaped bundles. Scale bars: 2 µm.

To determine whether MSRB3 plays a role in the early postnatal maturation and/or maintenance of hair cells and stereociliary bundles, we evaluated the gross morphology and cytoarchitecture of the organ of Corti in *Msrb3^−/−^* mutant mice in the first 3 weeks of life. We observed and catalogued the progressive structural abnormalities in the organ of Corti that occurred in the mutant mice compared to wild-type mice, leading up to and beyond the age at which the ABRs were recorded. The cochlear sensory epithelium in the mutant animals appeared normal at birth (Fig. S1). However, detectable abnormalities were seen by P4, including curving of the outer edges of the OHC stereociliary bundle (Fig. 3F,G, dashed line circles), suggesting that positions of peripheral stereocilia within a bundle and their rootlets are deviated from the normal positions within the cuticular plate, which could be due to altered rootlet to actin meshwork connections within the cuticular plate or impaired cuticular plate actin meshwork. These OHC changes were mostly restricted to the middle and basal turns of the cochlea; all inner hair cells (IHCs) and all apical-turn OHCs looked normal. By P10, however, phenotypic changes were seen throughout the cochlea when visualized with an anti-β-spectrin antibody, which, in control mice (Fig. 3D, arrows), uniformly labeled the cuticular plate except for the region of the kinocilium (Fig. 3). In nearly all mutant OHCs, however, several ‘β-spectrin-free’ zones near the cuticular plate edges were noted (Fig. 3L,P, arrows), as well as uneven distribution of β-spectrin within the OHC cuticular plate, likely indicating anomalies in the cuticular plate structure to which stereocilia rootlets are anchored (Pacentine et al., 2020) (Fig. 3). Although some hair cell death was obvious at this stage (Fig. 3J, asterisks), the cochlear sensory cells were largely preserved until ∼P16 (Fig. 3). By 3 weeks of age, the basal-most end of the cochlea was almost devoid of OHCs, but IHCs were still present. The organ of Corti in the remaining turns of the cochlea also frequently had missing or apoptotic hair cells at this stage (Fig. 3M-O,Q,R, asterisks; Fig. 4A, asterisks).

**Fig. 5.**
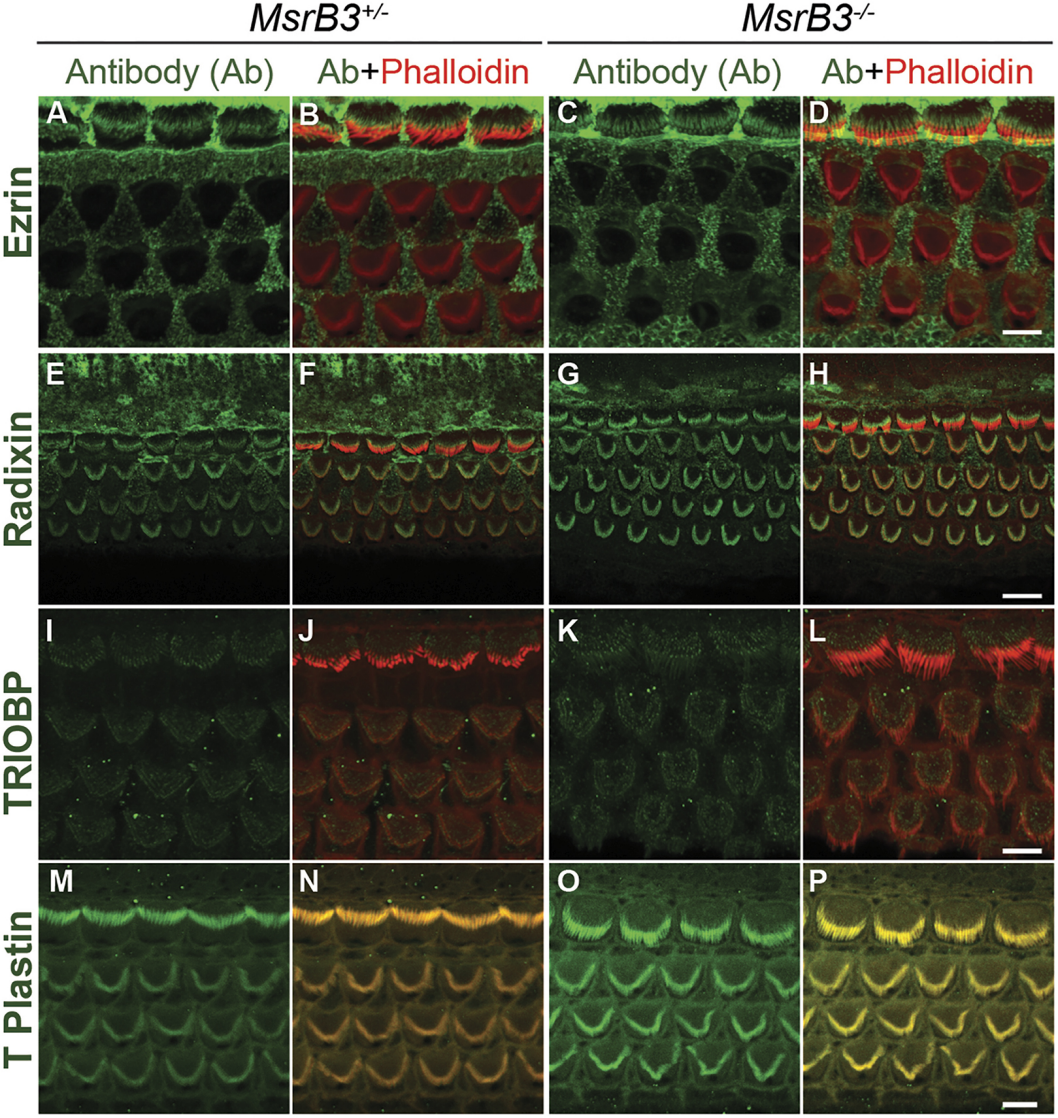
**Actin core-related proteins do not show an apparent change in their localization in the hair cells of *Msrb3^−/−^* mutant mice.** (A-P) Maximum-intensity projections of confocal *z*-stacks from the middle turn of the organ of Corti whole-mount cochleae labeled with antibodies against ezrin (A-D; green), radixin (E-H; green), TRIOBP4/5 (I-L; green) and T-Plastin (M-P; green), and counterstained by rhodamine-phalloidin (red), are shown for heterozygous and *Msrb3* mutant mice at P7. Scale bars: 5 µm (D,L,P; apply to A-C, I-K and M-O, respectively); 10 µm (H; applies to E-G).

Next, we evaluated the changes in the shape of the apical surface of hair cells and stereocilia bundles by scanning electron microscopy (SEM). At P4, the age at which abnormalities in hair bundle morphology were seen by phalloidin staining, the stereocilia bundles of OHCs in the basal turn of the cochlea of *Msrb3^−/−^* mice lost the characteristic ‘V’ shape (Fig. 4B, dashed line circle). These changes progressed rapidly, and, by P12, most of the stereocilia bundles in the middle and basal turns of the cochlea exhibited an ‘omega’ shape (Fig. 4B,C, arrows). The stereocilia were no longer upright but angled away from the basal body region (Fig. 4B,C, arrows). In some OHCs, the stereocilia were in various stages of dissolution, and the apical surfaces of most hair cells had a more rounded appearance compared to those of control cells (Fig. 4B,C, dashed lines indicate the shape of the hair cell surface in *Msrb3^−/−^* basal OHC compared to *Msrb3^+/−^* OHC at P12). The curving of the stereociliary bundles and the subsequent progressive collapse of the stereocilia suggested an anchoring defect of the stereociliary rootlets or overall destabilized actin structure. We aimed to determine structural alterations in stereociliary rootlets, after hearing onset, when stereociliary rootlets become well developed (P14-P21; Fig. S2). Transmission electron microscopy (TEM) imaging revealed seemingly intact rootlets, without any obvious structural alterations in the mutant *Msrb3^−/−^* hair cells at this age compared to control wild-type hair cells (Fig. S2A,B, arrows), although this observation cannot rule out a potential anchoring deficits due to loss of MSRB3 function. Occasionally, we observed some irregularities in the P14-P21 OHC cuticular plate density (Fig. S2D,F, arrows). We hypothesized that the stereocilia structural abnormalities noted in *Msrb3^−/−^* mice could be due to structural abnormalities in the cuticular plate actin meshwork or its connection to the stereociliary rootlets. This hypothesis was in agreement with our findings that, despite widespread defects in hair bundle shape, the localization of several apically expressed proteins – including ezrin, radixin, TRIOBP and T-Plastin (also known as PLS3) (Kitajiri et al., 2004) – remained unaltered in the P7 stereocilia and their rootlets (Fig. 5).

### Catalytically active MSRB3 can repolymerize actin *in vitro*

**Fig. 6.**
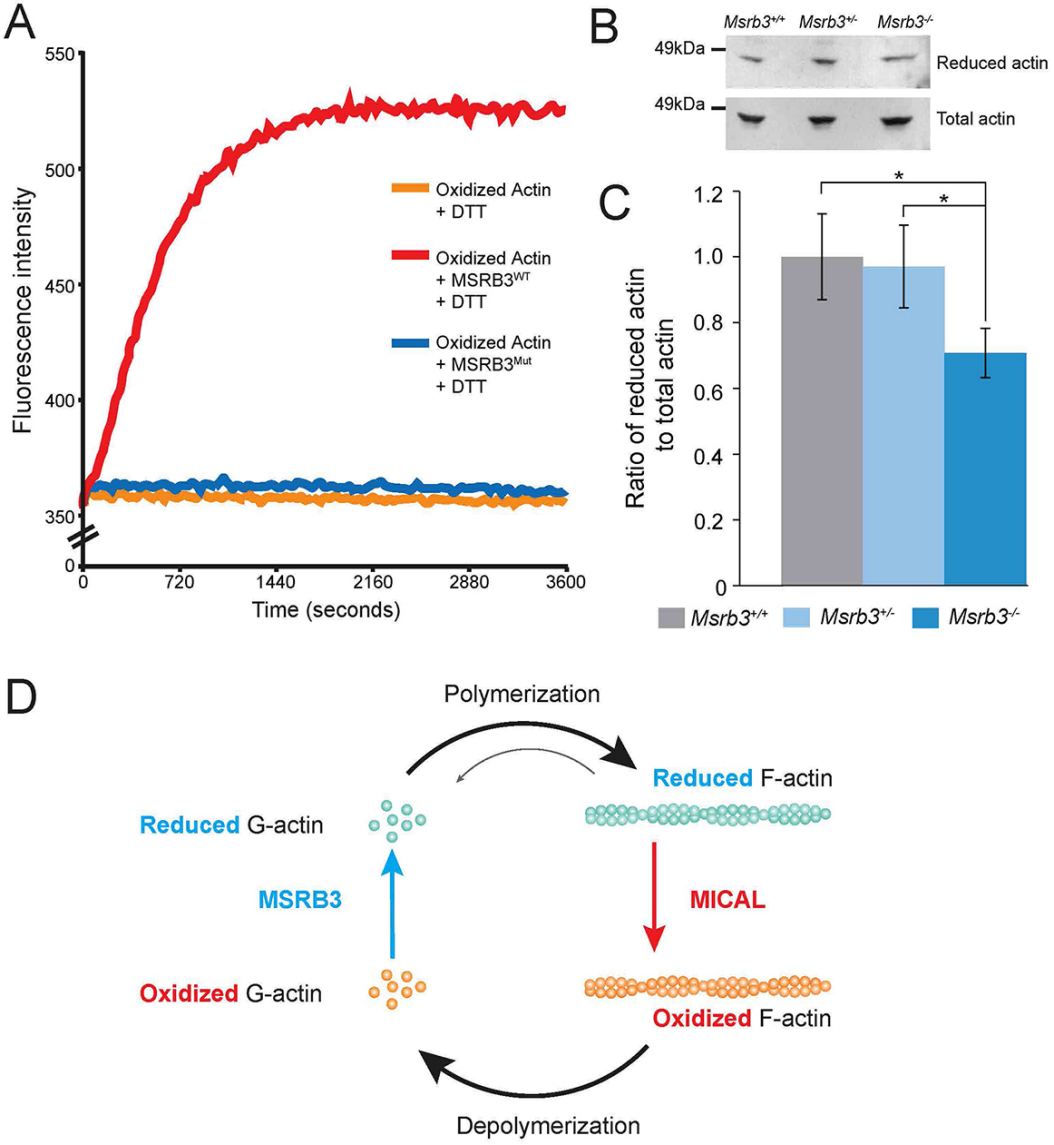
**MSRB3 promotes actin polymerization, and the absence of MSRB3 in mutant mice affects the reduced/oxidized actin ratio in the inner ear.** (A) Pyrene-labeled actin monomer was monitored for polymerization by assaying changes in fluorescence at 407 nm (excitation at 365 nm) in the presence of wild-type human MSRB3 (red, MSRB3^WT^) or deafness-associated protein MSRB3 p.Cys89Gly (blue, MSRB3^Mut^). As a control, fluorescence intensity of the preassembled pyrene-labeled actin was monitored in the absence of MSRB3 (orange). DTT, dithiothreitol. (B) Representative western blot showing the amount of reduced and total actin in the whole-inner ear tissue lysates from *Msrb3^−/−^* and control littermates (*Msrb3^+/−^* and *Msrb3^+/+^*) at P14, using antibodies specific for the reduced form of actin and total actin. (C) The ratio of reduced to total actin was calculated by using ImageJ to quantify the band densities shown in B. All data were normalized to the *Msrb3^+/+^* ratio (means±s.e.m.). This experiment was repeated independently four times with cochleae from three mice per condition. *Msrb3^−/−^* mutant mice showed a statistically significantly (*P<0.05, unpaired two-tailed Student's *t*-test) decreased ratio of reduced to total actin, suggesting a possible role for MSRB3 in the regulation of reduced actin to maintain the stereocilia structure in the inner ear. (D) Schematic of the role of MSRB3 in actin polymerization. MSRB3 activity is required to convert the oxidized G-actin back to reduced G-actin, which can be polymerized.

A prior study reported the role of MSRB1, another member of the MSR family, in facilitating actin assembly (Lee et al., 2013). MSRB1 catalyzes the reduction of methionines in actin that are oxidized by the microtubule-associated monooxygenase, calponin and LIM domain-containing proteins, MICAL1 and MICAL2 (Lee et al., 2013). Previous studies have attributed the oxidation of these methionine residues to inability of such actin monomers to polymerize to filamentous actin (F-actin) (Hung et al., 2010, 2011; Rouyere et al., 2022), a process crucial to stereocilia development, maintenance and function (Krey et al., 2023; Park and Bird, 2023). In a 2013 study, the authors concluded that the oxidation of actin by MICAL prevents filamentous actin assembly, while MSRB1 reverses the oxidation of these methionine residues, allowing actin repolymerization (Lee et al., 2013). To determine whether MSRB3 is also able to stereoselectively target oxidized methionine in actin, the repolymerization of MICAL-treated, pyrene-labeled actin was tested in the presence of full-length wild-type human MSRB3 (MSRB3^WT^) or a variant containing the p.Cys89Gly substitution (MSRB3^Mut^) that underlies human deafness DNFB74 (Ahmed et al., 2011). Whereas MSRB3^WT^ repolymerized oxidized actin, as measured by an increase in fluorescence intensity, loss of the catalytic p.Cys89 residue of MSRB3 abolished this activity (Fig. 6A). These *in vitro* biochemical studies support the notion that the hair bundle and cuticular plate abnormalities seen in the mutant mice are likely due to accumulation of oxidized methionines of G-actin molecules affecting actin dynamics, an essential feature of hair bundle function and maintenance.

### *Msrb3* mutant mice have a lower level of reduced actin

The hypothesis that the *Msrb3^−/−^* mutant mice accumulate G-actin with oxidized methionines was tested by quantifying the ratio of the reduced form of soluble actin to total soluble actin (reduced and oxidized). Using antibodies raised against reduced actin and total actin, the levels of the respective actin pools in the inner ear tissue samples of control and *Msrb3^−/−^* mutant mice were determined by western blotting. The amount of reduced actin in the inner ears of normal-hearing *Msrb3^+/−^* mice was found to be like that in wild-type animals. However, *Msrb3^−/−^* inner ears had significantly lower levels of reduced actin (Fig. 6B,C). Thus, we posit that the hair bundle defects seen in the *Msrb3* mutant mice are likely to be a result of elevated levels of oxidized actin, owing to the lack of the reducing activity of MSRB3 (Fig. 6D), affecting hair bundle dynamics and function in the cochlear sensory cells.

## DISCUSSION

MSRs are enzymes that repair damaged proteins by selectively reducing oxidized methionine residues (Kim and Gladyshev, 2007; Lee et al., 2009). Among the four paralogs (MSRA, MSRB1, MSRB2, MSRB3), so far, only MSRA and MSRB3 have been associated with hearing loss (Ahmed et al., 2011; Alqudah et al., 2018). *Msra* knockout mice show a progressive hearing loss, starting at the highest frequencies (Alqudah et al., 2018), and *MSRB3* variants in humans lead to prelingual bilateral profound hearing loss DFNB74 (Ahmed et al., 2011; Shafique et al., 2014; Richard et al., 2019). As has been observed previously in *Msrb3* exon 7 mutant mice (Kwon et al., 2014), our analysis of the cochlear phenotype of a *Msrb3* null mouse also revealed hair bundle abnormalities: a characteristic omega shape of the stereocilia bundle as early as P4 in the basal turn of the organ of Corti. In addition, *Msrb3* knockout mice exhibited progressive hair cell loss, starting with OHCs at the basal turn of the cochlear coil, until the sensory epithelium was completely degenerated by P60. Terminal deoxynucleotidyl transferase dUTP nick end labeling (TUNEL) immunoassays revealed that the hair cell loss observed in the mutant mice was due to apoptotic cell death (Fig. 4A). All these findings are consistent with a prior study utilizing *Msrb3* exon 7 deletion mutant mice (Kwon et al., 2014), except for the earliest signs of hair bundle dysmorphology. In contrast to reported abnormalities observed at P8 in *Msrb3* exon 7 deletion mutant mice (Kwon et al., 2014), we found stereocilia abnormalities in our *Msrb3* mutant mice at P4, which could be attributed to a complete null genotype or genetic background differences. In contrast to the congenic C57BL/6 background of our *Msrb3* mutant mice, the previously reported *Msrb3* deleted exon 7 mouse is on a 129/SvJ and C57BL/6 mixed background (Kwon et al., 2014). Furthermore, the C57BL/6 substrain used in the prior study was not described. Genetic deviations between C57BL/6 substrains can lead to phenotypic differences, including susceptibility to aminoglycoside-induced hearing loss and acquired hearing loss (Mekada and Yoshiki, 2021; Newton et al., 2025).

As the first sign of deficit observed in the mutant hair cells was the collapse of the stereociliary bundle observable by P4 in *Msrb3* null mice, we first focused on the three main components of the stereocilia that ensure its stability: the actin-core related proteins, the rootlets and the cuticular plate. Stereocilia are composed of an F-actin core that contains crosslinked, parallel actin microfilaments and actin cytoskeleton-associated proteins that have proven crucial for development, maintenance and stabilization of the stereocilia (Drummond et al., 2012). However, we observed no obvious differences in the expression of several of the actin core-associated proteins in mutant mice at P7 in unaffected hair bundles as well as in those with varying degrees of defects (Fig. 5).

At the tapered end of the stereocilium, the actin filaments form a rootlet that anchors a stereocilium in the actin-rich meshwork of the cuticular plate (Tilney et al., 1983; Tilney and DeRosier, 1986). Rootlets provide rigidity as well as flexibility to stereocilia, which are essential for optimal sensitivity of the hair bundle (Tilney et al., 1983; Tilney and DeRosier, 1986). Genetic studies have recently shed light on the central role of TRIOBP, an F-actin-bundling protein, in rootlet formation (Kitajiri et al., 2010; Katsuno et al., 2019). Interestingly, *Triobp* and *Ankrd24* mutants exhibit a stereociliary bundle phenotype similar to that of *Msrb3* knockout mice, where the hair bundle develops normally at first but, soon after the onset of hearing, the stereocilia fuse together, and the hair cells degenerate rapidly (Katsuno et al., 2019; Krey et al., 2022). In the *Triobp* isoform 4/5-deficient mouse models, the phenotype is explained by the absence of rootlets, which makes the stereociliary bundles fragile (Kitajiri et al., 2010; Katsuno et al., 2019). However, *Msrb3*-deficient mice did not exhibit any rootlet deficit at P14-P21 (Fig. S2), well beyond the age at which the hair bundle phenotypes are first seen. The similarity in the OHC bundle phenotype may be reflective of actin bundle integrity issues that are common to MSRB3, TRIOBP5 and ANKRD24 mutants. Interestingly, the omega-shaped bundle phenotype observed in MSRB3, TRIOBP5 and ANKRD24 mutants indicate some peripheral stereocilia anchoring defect that could be due to rootlet abnormalities or to altered connection of rootlets to the actin meshwork of the cuticular plate and/or abnormalities in the actin meshwork itself. The latter two reasons are likely explanations for *Msrb3^−/−^* hair cell phenotype.

We then questioned whether MSRB3 was involved in the maintenance and/or maturation of the stereocilia bundles. Indeed, hair cells of *Msrb3*-deficient mice seem to develop normally, initially, without any morphological aberration (Fig. S1) and exhibit normal uptake of the styryl dye, FM1-43, indicative of a functional mechanotransduction apparatus (Fig. 2C). However, morphological abnormalities in the stereocilia and hair cell degeneration are seen within the first 2 weeks of life, which accelerate with the maturation of the sensory epithelium and acquisition of hearing (Figs 3 and 4). These results suggest possible developmental abnormalities of stereocilia and/or the cuticular plate in which stereocilia are anchored in mutant mice. Our data show that MSRB3 is necessary for the structural integrity of the cuticular plate essential for normal stereociliary rootlet anchoring and maintenance of the stereociliary bundle.

The regulation of actin dynamics, through rapid turnover of depolymerized/polymerized actin, is essential for maintenance of stereocilia. Although two contradictory processes have been proposed – continuous depolymerization of the actin filaments at the base with repolymerization at the tip (Manor and Kachar, 2008), and continuous turnover at the tip of the stereocilia exclusively (Zhang et al., 2012; Narayanan et al., 2015; Drummond et al., 2015) – both mechanisms agree on the essential role of actin dynamics in the maintenance of the stereocilia. The transition between globular (G) and filamentous (F) actin and its dynamics are regulated by many actin-associated proteins. Among others, the role of MICAL, a monooxygenase, has been highlighted previously. In *Drosophila*, two methionine residues in actin (46th and 49th methionines) can be selectively oxidized by MICALs, inhibiting F-actin assembly (Hung et al., 2010, 2011). In humans, MICAL homologs were also found to regulate actin microfilaments and actin stress fibers (Giridharan et al., 2012), strengthening the implication of MICAL-dependent oxidation in regulation of actin dynamics. Lee et al. (2013) linked the activity of MICAL protein to two MSRB proteins: MSRB1 and MSRB2. Contrary to MSRA, these proteins can repolymerize G-actin that is previously disassembled by MICAL. These MSRB proteins work in a stereospecific manner, with MICAL regulating the actin assembly turnover. We hypothesized that MSRB3 has the same MICAL-antagonistic effect and promotes the reassembly of oxidized actin through its reduction. Using the test standardized for MSRB1 and MSRB2 previously (Lee et al., 2013), we demonstrated that MSRB3 protein is indeed able to repolymerize oxidized actin (Fig. 6A), indicating a role for MSRB3 in the regulation of actin dynamics in the hair cell cuticular plate, maintaining its integrity. Interestingly, MSRB3 harboring the most reported variant (p.Cys89Gly) associated with human deafness (Richard et al., 2019) DFNB74 is unable to repolymerize G-actin.

Although other MSR proteins are largely involved in oxidative stress protection in mammals (Moskovitz et al., 2001; Fomenko et al., 2009; Martinez et al., 2017), the protective role of MSRB3 in redox homeostasis has yet to be determined. We did not observe signs of major oxidative stress in the inner ear of the mutant mice compared to their control littermates (Fig. S3), as also previously reported by others (Kwon et al., 2014). Importantly, none of the other MSR family genes were dysregulated in the cochlea of P0 *Msrb3^−/−^* mice (Fig. 1B), suggesting a unique role for MSRB3 that cannot be compensated by MSR paralogs. Based on the MSRB3/MICAL antagonistic effect on actin dynamics, we speculated that MSRB3 might have a protective effect on actin molecules and reverse oxidative damage (Fig. 6D). In support of this, we found that the inner ears of knockout mice had a decreased ratio of reduced actin to total actin compared to that seen in wild-type mice, suggesting that, among all MSR proteins, MSRB3 is particularly critical for reversing oxidation of actin molecules in the inner ear. Taken together, these data support the role of MSRB3 in actin polymerization and dynamics, through its redox status. In the absence of MSRB3, less repolymerization of oxidized actin occurs, resulting in the disruption of the cuticular plate actin meshwork as well as altered stereocilia positions at the edges of a bundle, leading to the omega shape of the stereociliary bundle. In addition, using traditional TEM, it is not practical to observe the entire length and width of all rootlets of a hair cell stereocilia simultaneously, and we cannot rule out some rootlet abnormalities that could be revealed by a follow-up study on rootlet development in MSRB3 mutant mice using focused ion beam SEM with 3D reconstructions. In conclusion, this study establishes a specific role for MSRB3 in the inner ear. MSRB3 maintains hair cells through its redox mechanism to preserve normal actin dynamics in the cuticular plates and stereociliary bundles of the cochlear sensory cells, which is a prerequisite for the normal function of the these cells.

## MATERIALS AND METHODS

### Mutant mice and genotyping

The *Msrb3* knockout mice were recovered from a cryopreserved strain from KOMP/Velocigene. In this model, coding exons 2 to 7 were replaced by LacZ and Neo cassettes. To excise the Neo cassette, and avoid any potential phenotype linked to its presence, we crossed the mice acquired from Velocigene with a mouse line expressing the Cre recombinase under the *Zp3* promoter [C57BL/6-Tg(Zp3-cre)93Knw/J, The Jackson Laboratory, #003651). The females, from F1 generation, were then crossed with wild-type males (C57BL/6J), and the F2 generation was screened for excised alleles in both sexes. Mice were genotyped by multiplex PCR of both alleles using the following primers: 5′-GTGTCCTTGGATCTTCGAGC-3′ (forward), 5′-GAGGACGGTGAGGGTTTGTAAC-3′ (reverse, WT, amplicon 518 bp) and 5′-GTCTGTCCTAGCTTCCTCACTG-3′ (reverse, transgenic, amplicon 400 bp). Genotyping was performed using the following PCR protocol: 95°C for 2 min, 35 cycles (95°C for 30 s, 60°C for 30 s, and 72°C for 30 s), 72°C for 5 min. Amplimers were separated on a 2% agarose gel. All morphological assessments were performed with males and females, and no sex-related differences were detected.

### ABR and DPOAE

Hearing function was evaluated by ABR analyses at P16 of *Msrb3^+/+^* (*n*=7), *Msrb3^+/−^* (*n*=9) and *Msrb3^−/−^* (*n*=9) male mice. Mice were anesthetized with intraperitoneal injections of 2.5% Avertin (0.015 ml/g body weight). All recordings were done in a sound-attenuated chamber using an auditory-evoked potential diagnostic system (Intelligent Hearing Systems, Miami, FL, USA) with high-frequency transducers, as previously described (Giese et al., 2017). Responses to 50 µs duration clicks, and 8, 16 and 32 kHz tone bursts were recorded. Thresholds were determined in 5 or 10 dB steps of decreasing stimulus intensity, until waveforms lost reproducible morphology. The maximum sound intensity tested for each frequency was 110 dB SPL.

DPOAEs were recorded from *Msrb3^+/+^* (*n*=7), *Msrb3^+/−^* (*n*=9) and *Msrb3^−/−^* (*n*=9) male mice at P16 with an acoustic probe (ER-10C, Etymotic Research) using DP2000 DPOAE measurement system version 3.0 (Starkey Laboratory). Two primary tones, with a frequency ratio of *f*_2_/*f*_1_=1.2, where *f*_1_ represents the first tone and *f*_2_ represents the second, were presented at intensity levels *L*_1_=65 dB SPL and *L*_2_=55 dB SPL. *f*_2_ was varied in one-eighth octave steps from 8 to 16 kHz. DP grams comprised 2*f*_1_-*f*_2_ DPOAE amplitudes as a function of *f*_2_.

### X-galactosidase (X-gal) staining

β-galactosidase activity of the LacZ reporter gene product was detected by X-gal staining in tissue from the inner ear. Temporal bones were fixed for 20 min with 1% formaldehyde, 0.2% glutaraldehyde and 0.02% NP-40 diluted in phosphate-buffered saline (PBS), followed by washing for 1 h with 1× PBS containing 0.02% NP-40 and 2 mM MgCl_2_. Tissue was stained in the dark for 3 h at 37°C with X-gal solution [1 mg/ml X-gal, 5 mM K_3_Fe(CN)_6_, 5 mM K_4_Fe(CN)_6_, 0.1 M MgCl_2_ in PBS]. Temporal bones were washed and incubated overnight in 0.25 M EDTA and 2% paraformaldehyde solution. Inner ears were finely dissected and mounted onto a slide. Some of the temporal bones were cryopreserved in 30% sucrose, embedded in O.C.T.™ medium (Sakura Finetek USA), sectioned in 14 μm thick slices and mounted on glass slides. All images were acquired using a AxioVision Z1 system (Carl Zeiss, Inc.) fitted with an Apotome slide module with a 63×/1.4 NA Plan Apochromatic objective and a digital camera (AxioCam MRm; Carl Zeiss, Inc.).

### Immunofluorescence

Inner ears were fixed with 4% paraformaldehyde, overnight at 4°C and permeabilized with pre-block using 0.2% Triton X-100 in 10% heat-inactivated horse serum for 1 h. The paraformaldehyde-fixed cochleae were probed overnight with primary antibody and, after three washes, were probed with the secondary antibody for 1 h. For all hair cell body staining, rhodamine-conjugated phalloidin (Invitrogen) was used at 1:300 dilution to visualize the cuticular plate and hair bundles. Primary antibodies used were as follows: anti-MYO7A (Proteus, 25-6790) at 1:250, anti-β-spectrin (BD Biosciences, 612563) at 1:250, anti-MSRB3 (Sigma-Aldrich, HPA014432) at 1:50, anti-ezrin (Abcam, ab4069) at 1:50, anti-radixin (Abcam, ab52495) at 1:50 and anti-TRIOBP4/5 [Friedman laboratory (Kitajiri et al., 2010)] at 1:50. Alexa Fluor 488 goat anti-rabbit and Alexa Fluor 488 goat anti-mouse secondary antibodies (Invitrogen) were used at 1:500 dilution. DAPI was used at 20 µg/ml. After every secondary antibody incubation, the tissues were washed three times in PBS and mounted on slides using Vectashield^®^ mounting medium (Vector Labs) and viewed under a LSM meta 510 confocal microscope using a 63×/1.3 NA oil-immersion objective.

### SEM analysis

Inner ears were removed from animals after decapitation, and the cochleae were exposed. A small piece of cochlear capsule was removed from the cochlear apex, and the entire bulla was immersed in fixative (2.5% glutaraldehyde, 0.1 M sodium cacodylate containing 2 mM CaCl_2_ for 1.5 h). After three quick washes with 0.1 M sodium cacodylate buffer, the inner ears were post-fixed in 1% osmium tetroxide in 0.1 M sodium cacodylate buffer for 1 h at room temperature. The inner ears were washed three times with PBS before leaving in PBS containing 0.25 M EDTA for 2 days at 4°C. The samples were then microdissected in water to remove the stria vascularis, and the spiral ligament was cut off to expose the organ of Corti. The cochlear tissues were then dehydrated using an ethanol gradient, critical-point dried, sputter coated with gold and imaged on a scanning electron microscope (Hitachi SU3900). Where necessary, the images were adjusted for optimal brightness and contrast using Photoshop^®^ creative studio CS5.1 (Adobe).

### TEM

Inner ears of P14 and P21 *Msrb3* homozygous wild-type and mutant mice were dissected and fixed using 2.5% glutaraldehyde and 2% paraformaldehyde (Electron Microscopy Sciences) in 0.1 M phosphate buffer for 2 h at room temperature, washed in 1× phosphate buffer, decalcified in 100 mM EDTA in 1× phosphate buffer for 48-72 h at 4°C, washed in phosphate buffer, dehydrated in increasing concentrations of ethanol, poststained in 1% osmium tetroxide and embedded into epon-812 resin (Electron Microscopy Sciences) as previously described (Tilney and DeRosier, 1986). Ultrathin sections of ∼70-90 nm were cut using ultracut UCT-7 (Leica), post-stained with 1% uranyl acetate (Electron Microscopy Sciences) and lead citrate (Sigma-Aldrich) and visualized using a JEOL1200 transmission electron microscope (JEOL).

### Western blotting

Inner ears were dissected from two mice for each of the indicated genotypes at P14. The tissues were homogenized in 200 µl lysis buffer [0.05 M Tris-HCl pH 7.5, 150 mM NaCl, 0.1% NP40 and cocktail protease inhibitor (Sigma-Aldrich)] and sonicated to sheer the DNA followed by centrifugation at 10,000 ***g*** for 20 min at 4°C to pellet the cellular debris. The supernatants were collected in fresh tubes, and ¼ volume of 4× Laemmli sample buffer without any reducing agent was added. The samples were heated for 12 min at 70°C and centrifuged, before resolving 25 µl of each sample on a 10% SDS-PAGE gel. After electrophoresis, the samples were electrophoretically transferred onto nitrocellulose membranes by the semi-dry western blotting method. The blots were pre-blocked in 5% low-fat dried milk powder in Tris-buffered saline containing 0.05% Tween 20 before incubating with pan actin antibodies (Cytoskeleton Inc., AAN01) at 1:1000 and reduced actin antibodies (described in Lee et al., 2013) at 1:1000. After three washes, the blots were incubated with horseradish peroxidase-conjugated goat anti-rabbit secondary antibody (GE Healthcare) at 1:1000, and the signals were detected using an enhanced western blotting kit (Piercenet). Alongside all the samples, a western blotting molecular standard marker was used to determine approximate molecular masses.

### Semi-quantitative expression studies (RT-PCR)

RNAs from inner ear were extracted at P0 from *Msrb3* wild-type, heterozygous and homozygous mice using a Ribopure kit (Ambion). A SMARTScribe Reverse Transcriptase kit (Clontech) was used to generate cDNAs, and SYBRgreen technology (Qiagen) was used to perform quantitative PCR using gene-specific primers. *Gapdh* amplification was used to normalize the samples.

### Actin polymerization assay

Repolymerization of the actin, depolymerized by oxidation, was examined with modification as described previously (Hung et al., 2011). For this assay, we used pyrene-labeled actin, which is a modified G-actin generated by covalent conjugation with a fluorescent pyrenyl group at the C-terminus of the actin molecule. Briefly, pyrene-labeled actin was oxidized by treatment with hydrogen peroxide and then prepared for the polymerization assay by transferring it to polymerization buffer (Cytoskeleton, Inc.). This oxidized pyrene-labeled actin, with or without MSRB3^WT^ or MSRB3^p.Cys89Gly^ (MSRB3^Mut^), was monitored for changes in fluorescence intensity to assess polymerization in the presence of dithiothreitol.

## Supplementary Material

10.1242/dmm.052194_sup1 Supplementary information
